# Nanodiamond Decorated PEO Oxide Coatings on NiTi Alloy

**DOI:** 10.3390/nano13182601

**Published:** 2023-09-20

**Authors:** Karlis Grundsteins, Kateryna Diedkova, Viktoriia Korniienko, Anita Stoppel, Sascha Balakin, Kaspars Jekabsons, Una Riekstina, Natalia Waloszczyk, Agata Kołkowska, Yuliia Varava, Jörg Opitz, Wojciech Simka, Natalia Beshchasna, Maksym Pogorielov

**Affiliations:** 1 Institute of Atomic Physics and Spectroscopy, University of Latvia, 3 Jelgavas St., LV-1004 Riga, Latvia; karlis.grundsteins@gmail.com (K.G.); katefei93@gmail.com (K.D.); vicorn77g@gmail.com (V.K.); kaspars.jekabsons@lu.lv (K.J.); una.riekstina@lu.lv (U.R.); 2 Biomedical Research Centre, Sumy State University, 2 Rymskogo-Korsakova St., 40007 Sumy, Ukraine; yuliia.varava@gmail.com; 3 Fraunhofer Institute for Ceramic Technologies and Systems IKTS, 01109 Dresden, Germany; anita.stoppel@ikts.fraunhofer.de (A.S.); sascha.balakin@ikts.fraunhofer.de (S.B.); joerg.opitz@ikts.fraunhofer.de (J.O.); natalia.beshchasna@ikts.fraunhofer.de (N.B.); 4 Faculty of Chemistry, Silesian University of Technology, 9 Strzody St., 44-100 Gliwice, Poland; natalia.szulc@polsl.pl (N.W.); agatkol653@student.polsl.pl (A.K.)

**Keywords:** NiTi, nanodiamond, plasma electrolytic oxidation, biocompatibility, surface treatment

## Abstract

Cardiovascular diseases (CVDs) remain a leading cause of death in the European population, primarily attributed to atherosclerosis and subsequent complications. Although statin drugs effectively prevent atherosclerosis, they fail to reduce plaque size and vascular stenosis. Bare metal stents (BMS) have shown promise in acute coronary disease treatment but are associated with restenosis in the stent. Drug-eluting stents (DES) have improved restenosis rates but present long-term complications. To overcome these limitations, nanomaterial-based modifications of the stent surfaces have been explored. This study focuses on the incorporation of detonation nanodiamonds (NDs) into a plasma electrolytic oxidation (PEO) coating on nitinol stents to enhance their performance. The functionalized ND showed a high surface-to-volume ratio and was incorporated into the oxide layer to mimic high-density lipoproteins (HDL) for reverse cholesterol transport (RCT). We provide substantial characterization of DND, including stability in two media (acetone and water), Fourier transmission infrared spectroscopy, and nanoparticle tracking analysis. The characterization of the modified ND revealed successful functionalization and adequate suspension stability. Scanning electron microscopy with EDX demonstrated successful incorporation of DND into the ceramic layer, but the formation of a porous surface is possible only in the high-voltage PEO. The biological assessment demonstrated the biocompatibility of the decorated nitinol surface with enhanced cell adhesion and proliferation. This study presents a novel approach to improving the performance of nitinol stents using ND-based surface modifications, providing a promising avenue for cardiovascular disease.

## 1. Introduction

Cardiovascular diseases (CVD) are responsible for more than 25% of deaths in the European population, with >60 million potential years of life lost due to CVD in Europe annually [1,2]. Atherosclerosis (fibrofatty lesions in the artery wall) is the leading cause of CVD and leads to most myocardial infarctions and many strokes, as well as peripheral artery disease [3]. Atherosclerotic plaque leads to the narrowing of medium-size vessels that cause inadequate perfusion of the heart, brain, or other affected tissues. Despite the high efficacy of statin drugs in the prevention of atherosclerosis, they are not effective in decreasing the plaque size and vascular stenosis level. Since 1994, bare metal stents (BMS) have been approved for application in patients with acute coronary disease, such as myocardial infarction [4]. BMSs are made of stainless steel, cobalt–chromium alloys (Co-Cr), or nickel–titanium alloys (nitinol) [5] and have shown remarkable results in acute coronary disease treatment and the rehabilitation of patients. But, in approximately 15–20% of all implanted BMS stents, patients required reintervention 6–12 months after the first implantation due to restenosis [6,7]. To prevent the formation of thrombus in the stent, the deposition of antiproliferative drugs in the concept of drug eluting stents (DES) with time-ordered drug release [8]. Despite the remarkable clinical effectiveness of in situ restenosis, the long-term presence of nonbiodegradable materials in DES leads to late complications, such as thrombosis, neointimial hyperplasia, and chronic inflammation [9]. Based on the review [4], the failure of DES for long-term clinical use is associated with the following factors: (1) permanent polymeric coating materials, (2) metallic stent platforms, (3) non-optimal drug release conditions, and (4) factors that have recently been supposed to be contributing factors, such as polymer degradation products, metal ions due to erosion, and the degradation of metals and their alloys that make up some stents as metal frameworks. To overcome the existing limitation, biodegradable scaffolds with an eluting polymer coating are proposed that have some clinical advantages. However, retrospective studies demonstrate the risk of acute strut fracture as a result of insufficient mechanical strength compared to metallic DES, increased rates of early thrombosis, and a poor degradation and resorption profile [10].

Recent progress in nanomaterial design opens up new perspectives in the treatment of cardiovascular disease, including modification of the surface of the stent. I. Cicha and coauthors summarized the possible technologies for stent modification, which include, primarily, the impregnation of nanoparticles in the polymer layer of DES that prolongs the effectiveness but does not eliminate the problem after polymer degradation [11]. Some commercial stents have realized the concept of BMS-based drug-filled reservoirs that reduce the area and duration of contact between tissue and polymer [12]. However, all these technologies depend on the release of antiproliferative drugs that have a limit in time and no excide period in one year with later adverse effects typical for both BMS and DES. As a reliable alternative, non-pharmacological solutions to address device restenosis; therefore, coating with silicon carbide, due to its semiconductor nature, demonstrates a reduction in inflammatory and thrombogenic responses [13]. The drug-coated version (sirolimus) of this technology is also available and shows significant clinical advantages [14]. The main target of this technology is the continuous protection of stents from restenosis after the drug elution and coating degradation are completed. To achieve this concept, new nanoparticles, including detonation nanodiamonds (ND), were tested, which demonstrate some critical advantages for stent surface modification. Some studies have already shown that diamond-shaped carbon-coated nitinol stents demonstrate a reduction in neointimal hyperplasia compared to untreated stents [15].

NDs are bioinert carbon-based nanomaterials [16]. Since the surface of ND is facile to functionalize, it is used in various biomedical applications, such as bioimaging, anticancer therapy, gene delivery, and bone tissue engineering [17]. Additionally, they are used to promote cell growth and inhibit S. aureus colonization on Ti-based implants [18], as well as an additive in hydroxyapatite coatings [19]. Here, functional NDs are used as additives in the oxide layer of nitinol to enhance reverse cholesterol transport (RCT). In nature, high-density lipoproteins (HDL) composed of apolipoprotein A1 and phospholipids surrounding a core of cholesteryl esters and triglycerides are responsible for the removal of excess cellular cholesterol from peripheral tissues and transporting it back to the liver in the atheroprotective process called RCT [20]. RCT is believed to be a protection mechanism against cardiovascular diseases. Increasing HDL levels offers a promising therapeutic strategy to prevent and possibly reverse atherosclerosis by increasing RCT [21]. The main purpose of the modified ND is to mimic the HDL–cholesterol interaction, which naturally occurs in human blood during RCT. NDs will be incorporated into the nitinol surface and facilitate cholesterol efflux.

ND surface modification is widely used for the improvement of Ti-based bone implants to control wear resistance [22] and improve bone cells activation [23]. Some recent studies demonstrated enhanced endothelial vascular cell attachment and proliferation in culture with ND [24]. The chemical vapor deposition (CVD) [25], dip coating technique [26], and direct deposition [27] of ND to implant surface are widely used with some limitations, e.g., non-uniform ND distribution and pure adhesion. The plasma electrolytic oxidation (PEO) technique could overcome the limitations of the methods and provide a strong adhesion and uniform distribution of ND to the metal surface [28]. Our previous research demonstrated a successful application of PEO for the incorporation of Ag and ZnO nanoparticles in the oxide layer of TiAlV and ZrNbTi alloys [29,30,31]. In contrast to Ti-based alloy, PEO of nitinol is a challenging process because the formation of oxide layers by PEO on NiTi alloy is relatively difficult due to the large amount of nickel, which does not support the growth of the anode layer. The TiO_2_ layer can form a biologically inert stent surface, reducing the risk of stent thrombosis. In addition, by using a layer of titanium oxide, the metal stent can be protected from direct contact with the inner surface of the vessel after the end of drug release; this prevents harmful ions from entering the metal stent [32]. The following are used as electrolytes: phosphoric acid—H_3_PO_4_ (galvanostatic conditions, current density 10–30 A/m^2^, temperature below 0 °C) [33], sodium phosphate—Na_3_PO_4_, sodium aluminate and sodium hypophosphite—NaAlO_2_/NaPO_2_H_2_ (in both cases, potentiostatic conditions, voltage 100–300 V, temperature 10 °C) [32], and sodium sulfate (VI) and sodium hydroxide-Na_2_SO_4_/NaOH (alternating current source, voltage 80 V, frequency 50 Hz) [33]. By manipulating the process conditions, surfaces with improved or different biofunctionalities can be produced for specific clinical applications. The PEO layer increases the hardness of the NiTi alloy, increases its wear and friction resistance, and protection against corrosion. The PEO process can also enhance the adhesion of the oxide surface to the NiTi alloy substrate. This is very important for applications where a strong and durable protective layer is required [32,33,34]. Some research has demonstrated the success of nitinol in a sodium sulfate solution [33] and phosphoric acid [35] for biomedical applications, but there are no data about ND incorporation into NiTi PEO coating. Our research demonstrated the first application of ND for the decoration of nitinol PEO coating with the aim of producing a highly biocompatible surface with protection against reversing atherosclerosis. Here, we demonstrate the whole process of ND functionalization, nitinol PEO coating with ND incorporation, and the detailed structural and chemical investigation and biological assessment.

## 2. Materials and Methods

### 2.1. Nanodiamonds Functionalization

We used an arylation technique to provide nanodiamond functionalization to biomedical applications. The arylation of pristine ND was performed in the following three steps: 1—ND oxidation by thermal annealing, 2—hydroxylation using borane reduction treatment, and 3—arylation via addition of aminobenzoic acid and amyl nitrite. Thermal annealed ND will be named as ND-COOH, hydroxylated ND as ND-OH and arylated ND as ND-Aryl.

To synthesize ND-COOH, high-temperature air oxidation was used. Oxidation was carried out in an HTK 17/17 furnace by annealing in ambient air at 415 °C for 4.5 h with a heating rate of 20 K/min. Several ceramic plates were used to allow a high rate of exposure to the ND surface. Subsequently, the ND samples were pooled. Approximately 800 mg of ND were placed on each tray (diameter: approximately 6 cm).

In order to increase the number of -OH groups on the ND surface, hydroxylation was carried out according to the borane reduction protocol. In summary, by using borane as a reducing agent, the carbonyl groups on the ND surface are converted into alcohol functions. In a round bottom flask, 2 g of ND-COOH were suspended in 120 mL of dry THF. In total, 5 mL of 1 M BH3·THF was added dropwise under stirring conditions. The mixture was boiled under reflux for 24 h in an inert environment (nitrogen) at 64 °C. After being cooled to room temperature (RT), the mixture was hydrolyzed with 2 M HCl until hydrogen evolution stopped and no distinct air bubbles were visible. The solid product was isolated using centrifugation. Subsequently, the ND-OHs were washed four times with ultrapure water and three times with acetone. Each washing cycle included a 10 min resuspension supported by ultrasound and a 30 min centrifugation at 15,000 rpm.

Aromatic groups are bound to the surface by a diazonium salt reaction. ND arylation was performed by adding 10 g of aminobenzoic acid and 5 mL of amyl nitrite to 10 mL of a 150 mg/mL aqueous ND of 150 mg/mL stirring at 80 °C for 15 h. Amyl nitrite was used for the in situ generation of diazonium salts. The aminobenzoic acid-derived diazonium salt was conjugated to the ND via the click chemistry method. Five washing cycles in ddH_2_O and 15 in acetone were required to obtain a transparent supernatant after centrifugation and to ensure that excess chemicals were removed. All ND samples were dried overnight using a vacuum desiccator after surface modification.

### 2.2. Nanodiamond Assessment

Fourier transmission infrared spectroscopy (FTIR) (FTIR-6100, PerkinElmer, Rodgau, Germany) was used to characterize the ND surface. KBr pellets were prepared by mixing 2 mg of ND and 100 mg of KBr (Sigma-Aldrich Chemie GmbH, Taufkirchen, Germany). Infrared absorption spectra were recorded in the range of 450–4000 cm^−1^ with four scan cycles and a resolution of 2 cm^−1^. Three measurements were made for each sample and the mean value was determined for further analysis.

Depending on the interplay of electrostatic and van der Waals interactions, ND can aggregate in aqueous media or exist as homogeneous dispersions. To investigate and further use of the ND, we first tested the stability in two media (acetone and water). For this purpose, the NDs were suspended in the respective media by ultrasound and then calmly positioned.

Nanoparticle tracking analysis (NTA) (ZetaView PMX120, Particle Metrix GmbH, Inning am Ammersee, Germany) was used to determine the particle size distribution. In total, 1 mg of ND was mixed with ddH_2_O and sufficiently diluted. Measurements were recorded at RT and neutral pH = 7. The wavelength of the laser was 488 nm.

### 2.3. Cytotoxicity of ND

To provide a calculation of the effective ND concentration of the surface treated with PEO, its cytotoxicity was evaluated on human dermal fibroblasts obtained from the research group of the LU MF pharmacy program over 3 days. ND was sterilized with 70% ethanol for 30 min and washed with phosphate buffered saline (PBS) 3 times by centrifuged for 5 min at 1200 RPM. Dermal fibroblasts were grown in a T75 cell culture flask in Dulbecco’s modified Eagle medium/nutrient mixture F-12 (DMEM/F-12) with L-glutamine containing 100 units of mL^−1^ penicillin, 100 µg mL^−1^ streptomycin, 2.5 µg mL^−1^ amphotericin B, and 10% fetal bovine serum under standard culture conditions with medium renewal every 2 to 3 days. Cells were seeded in 96-well plates at a density of 10 × 10^3^ cells per well overnight. Subsequently, 100 µL ND was added to the plate at concentrations ranging from 20 mg/mL to 0.039 mg/mL with a dilution of 1:2. CCK-8 (Dojindo Laboratories, Tokyo, Japan) was added to each well of 10 µL (10% of the volume of medium) and incubated for 2 h at 37 °C after the first day. After incubation, 100 μL of medium from each well was transferred to a fresh 96-well plate. The absorbance was measured using a Tecan Infinite M200 Pro instrument (Tecan Trading AG, Männedorf, Switzerland) at 450 nm with reference to 620 nm wavelengths. The turbidity of the ND solution can affect CCK-8 absorbance and we used a cell-free ND solution as a negative control to correct the optical density results.

### 2.4. Surface Modification of the NiTi Alloy

Samples of the NiTi alloy (Bimotech, Wrocław, Poland) with a diameter of 8 and a height of 4 mm were subjected to plasma electrochemical oxidation (PEO). Before the process, they were sanded with SiC sandpaper with a gradation of 320, 600, and 800. Then they were degreased in isopropyl alcohol in an ultrasonic cleaner for 5 min. Thus, they were subjected to PEO.

The PEO process was performed in a cooled cell, where the sample was the anode and the stainless steel sheet was the cathode. The temperature of the electrolyte was 10 °C. The cell was powered by a Kikusui PWR 800H high-voltage power supply (Kikusui, Yokohama, Japan). Concentrated phosphoric acid H_3_PO_4_ was used as an electrolyte. The process was carried out at a current density of 100 mA/cm^2^, using limiting voltages of 50 (PEO1), 60 (PEO2), or 70 V (PEO3). The process was carried out in the galvanostatic mode until the limiting voltage was reached, and then in the constant voltage mode. The processing time was 5 min.

The ND suspension with an initial concentration of 0.04 mg/mL was deposited on the surface of the PEO-nitinol discs using a drop coating deposition method followed by drying at room temperature overnight. This procedure was repeated twice to obtain ND-PEO-nitinol disks with nanodiamond concentrations of 0.08 mg/mL.

After the PEO process and the application of the nanodiamond suspension, the NiTi alloy samples were analyzed for morphology and chemical composition using a scanning electron microscope (SEM) equipped with an X-ray energy dispersive spectrometer (EDX) (Phenom ProX; ThermoFischer Scientific, Watham, MA, USA). The samples after the PEO process were also subjected to cross-sectional analysis, which was observed using the Phenom ProX microscope. The surface contact angle of the samples was also determined using the OCA15 goniometer (DataPhysics Instruments, Filderstadt, Germany) with measurements of three samples in each group. The roughness was measured with the use of SEM and dedicated software.

### 2.5. Biocompatibility of Nitinol-PEO-ND 

The cytotoxicity and biocompatibility of ND-PEO-nitinol disks were determined on human dermal fibroblasts (LU MF pharmacy program research group). Disks were sterilized in 70% ethanol for 30 min and washed with phosphate-buffered saline (PBS) 3 times for 5 min to remove any ethanol residues. Cells were cultured in Dulbecco’s modified Eagle medium/nutrient mixture F-12 (DMEM/F-12, Gibco, USA) supplemented with 100 units mL^−1^ penicillin, 100 µg mL^−1^ of streptomycin, 2.5 µg mL^−1^ of amphotericin B (Gibco, ThermoFischer Scientific, Watham, MA, USA) and 10% fetal bovine serum under standard humidified air containing 5% CO_2_ at temperature 37 °C with medium renewal every 2–3 days. The samples were placed in a 48-well plate and dermal fibroblasts were seeded at a density of 104 cells per well. After 24 h, 10 µL (10% of the volume of medium) of CCK-8 (Dojindo Laboratories, Tokyo, Japan) was added to each well and incubated for 2 h at 37 °C. The optical absorbance was measured at 450 nm with reference to 620 nm wavelengths by using a Tecan Infinite M200 Pro instrument (Tecan Trading AG, Männedorf, Switzerland). The CCK-8 assay was repeated on the third and seventh days.

On the seventh day, the disks were washed twice with PBS, fixed with 4% formaldehyde (Saint Louis, Missouri, USA) for 10 min, and penetrated by 0.1% Triton X-100 in PBS with 1% BSA. The nuclei of the dermal fibroblasts were stained with Hoechst 33342 (ThermoFischer Scientific, Watham, MA, USA) diluted 1:1000 and the cell cytoskeleton was stained with ActinRed 555 (ThermoFischer Scientific, Watham, MA, USA). The samples were analyzed with the Nikon Eclipse TI fluorescence microscope (Nikon, Sendai, Japan) in the DAPI and TRITC channels.

## 3. Results

### 3.1. ND Characterization

**Figure 1:**
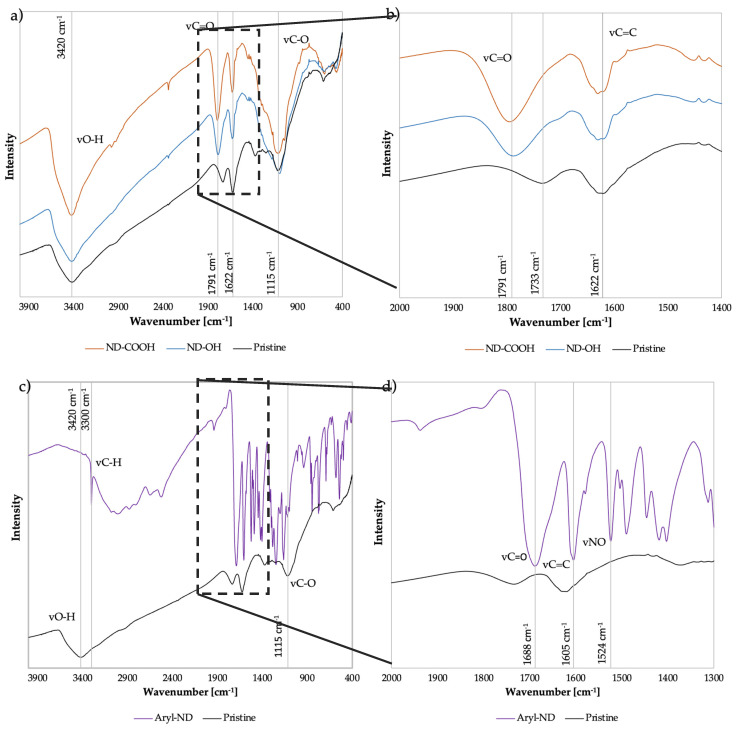
(**a**) Infrared spectra of detonated (black), oxidized (orange), and hydroxylated (blue) ND. (**b**) The right-hand boxes show a closer look at the FT-IR spectra in the range of 2000–1400 cm^−1^. (**c**) Infrared spectra of detonated (black) and arylated (red) ND. (**d**) shows a closer look at the FT-IR spectra in the range 2000–1300 cm^−1^.

Surface characterization of the ND was performed by FTIR to identify functional groups. Figure 1 plots the IR transmittance of pristine, carboxylated, hydroxylated, and arylated ND against the wavenumber. At 1791 cm^−1^ oxidized and hydroxylated ND show carbonyl stretching vibrations νC=O, where the C=O can be part of aldehyde, ketone, ester, carboxylic acid, anhydride, or cyclic ketone groups. Figure 1b shows the FTIR spectra in the range of 2000–1400 cm^−1^, where the relative increase in the intensity of the carbonyl band at 1791 cm^−1^ and the decrease in the C=C group at 1622 cm^−1^ is visible in oxidized (ND-COOH) and hydroxylated ND (ND-OH) compared to ND. As can be observed, the intensity of carbonyl stretching vibrations νC=O increases at 1791 cm^−1^, while the sp2 carbon νC=C decreases for both ND-COOH and ND-OH compared to ND. The dry oxidation method leads to the formation of acid anhydrides on the ND surface, which can be seen by the respective red shift of the νC=O bands in the FTIR spectra. Here, the shift from the νC=O band to a higher wavenumber from 1733 cm^−1^ (pristine ND) to 1791 cm^−1^ (ND-COOH and ND-OH) is observed based on dry oxidation, where the C=O bond of acid anhydrite is expected to appear around 1800 cm^−1^ and of carboxylic acid at 1760 cm^−1^ [36,37].

The region between 900 and 1500 cm^−1^ exhibits broad absorption and is also referred to as the ‘fingerprint’ region. This IR region is notoriously difficult to interpret in nanodiamonds because many components of the interior of the diamond and the surface overlap. In this region, deforming O-H and C-O-C stretching vibrations, epoxy C-O stretching vibrations, C-C stretching vibrations, amide C-N stretching, and C-N-H deformation vibrations overlap, along with NO_2_, SO_2_, and others.

After the reduction with borane, the concentration of hydroxyl groups should be increased. Because strongly bound water sits on the diamond surface, it is difficult to detect hydroxyl groups in the spectrum; moreover, even if the diamonds are excessively dried under vacuum, the samples are not anhydrous.

Figure 1c compares the infrared spectra of arylated ND and DND. At 3420 cm^−1^, DND exhibits νO-H stretching vibrations, as well as at 1115 cm^−1^, which corresponds to νC-O. These oscillations are not clearly seen in arylated ND. Aryl-ND shows a strong band at 3300 cm^−1^, which belongs to νC-H. Aromatic compounds are characterized by this band, but are often masked by the bands of other groups [38]. Figure 1d shows a closer look at the FT-IR spectra in the region of 2000–1300 cm^−1^, where the aryl-ND has aromatic carboxylic acid νC=O at 1688 cm^−1^ and νC-OH at 1490 cm^−1^ due to the functional aryl groups (benzoic acid) onto the ND [39,40]; moreover, nitrogen dioxide vibrations νNO_2_ are observed at 1524 cm^−1^, which is related to the amyl nitrite employed during the ND surface modification [40]. 

**Figure 2:**
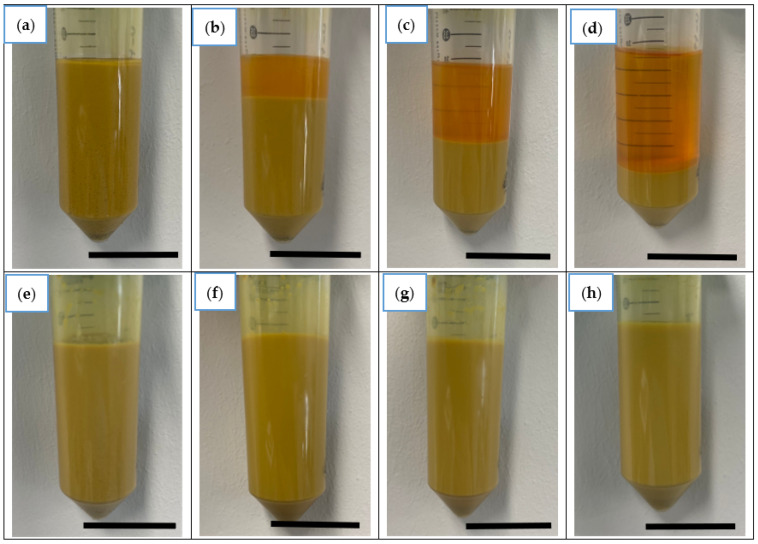
Experimental setup to study the stability of ND-Aryl in (**a**–**d**) acetone and (**e**–**h**) distilled water directly after suspension, after 2 min, 30 min, and 10 h (from left to right). Scale = 2 cm.

The colloidal stability experiment demonstrated that after only two minutes in acetone, the ND separated from the solution and formed a mixture until it settled, as shown in Figure 2a–d. Thus, it can be concluded that ND-Aryl does not exhibit stability in acetone. In distilled water, the ND formed a homogeneous and stable dispersion even after hours (Figure 2e–h).

**Figure 3:**
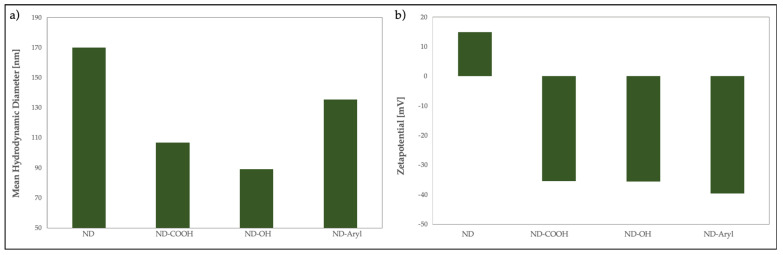
(**a**) Particle size distribution and (**b**) zeta potential by means of DLS for ND, ND-COOH, ND-OH, and ND-Aryl.

To ensure a high functionalization rate, NDs with a high surface-to-volume ratio are required. Accordingly, NTA was performed to evaluate the hydrodynamic diameters of the modified ND. Figure 3 shows the sedimentation rows, particle size distribution, and zeta potential of pristine ND, ND-COOH, ND-OH, and ND-Aryl, respectively. 

The pristine ND exhibits a mean hydrodynamic diameter of 170 nm. The modified NDs show mean sizes larger than those of the pristine ND, where ND-COOH has a mean diameter of 106 nm, ND-OH of 89 nm, and ND-Aryl of 135 nm. According to the manufacturer, NDs have a particle size of 4–5 nm. NDs form larger agglomerates due to different types of intermolecular interactions, such as van der Waals, dipole–dipole, and hydrogen bonding. Oxidation and hydroxylation lead to homogenized ND with a large number of oxygen-containing surface groups, which reduces their agglomerate size. ND-Aryl aggregates are smaller in comparison to those of pristine ND and larger than those of ND-OH. This phenomenon can be explained on the added functional groups (4-carboxyphenyl), which increases the hydrodynamic diameter. 

Pristine ND show a positive zeta potential of +15 mV due to the manufacturer’s specification. ND-COOH has a zeta potential of −36 mV, ND-OH of −35 mV, and ND-Aryl of −40 mV. Surface functionalization led to a negative zeta potential due to oxygen-rich surface groups. When the absolute value of the zeta potential is above ±30 mV, the particles in the suspension repel each other and form stable suspensions.

The sedimentation row provides a clearer picture of the stability of the suspension. The pristine NDs are unstable and precipitate, where ND-OH, ND-OH, and ND-Aryl display a stable suspension.

To obtain a high surface-to-volume ratio, which is necessary both for homogeneous coatings and for functionalization processes, stable ND suspensions with the smallest possible particle size must be obtained; therefore, deagglomeration is necessary.

### 3.2. Nitinol PEO Surface Characterization

**Figure 4:**
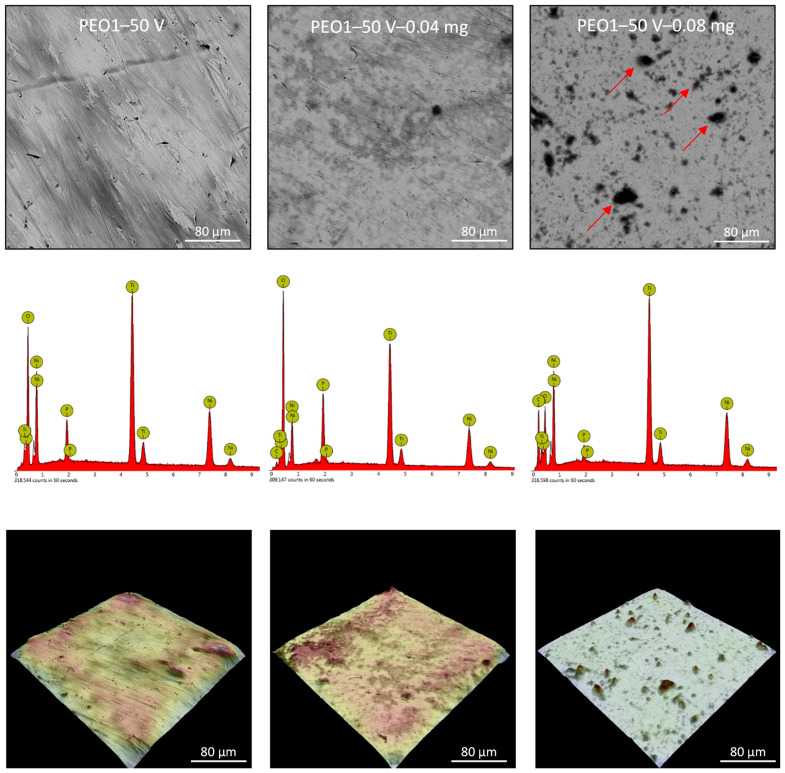
Surface morphology and chemical composition of the PEO1 sample, and PEO1 samples with nanodiamonds (0.04 or 0.08 mg); SEM—upper row; EDX—middle row; 3D reconstruction—lower row; red arrows—particles agglomerates.

**Figure 5:**
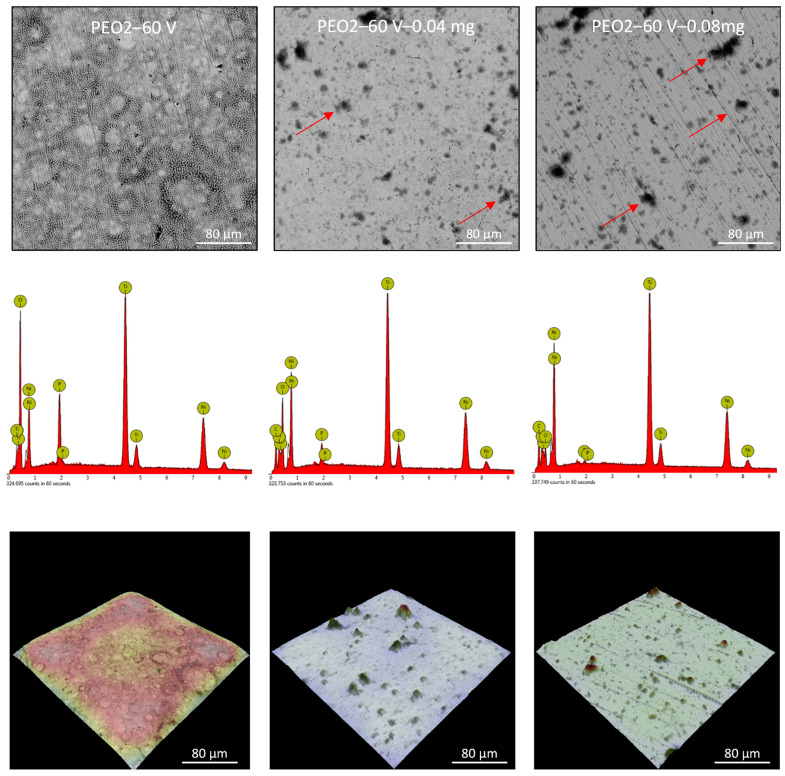
Surface morphology and chemical composition of the PEO2 sample, and PEO2 samples with nanodiamonds (0.04 or 0.08 mg); SEM—upper row; EDX—middle row; 3D reconstruction—lower row; red arrows—particles agglomerates.

**Figure 6:**
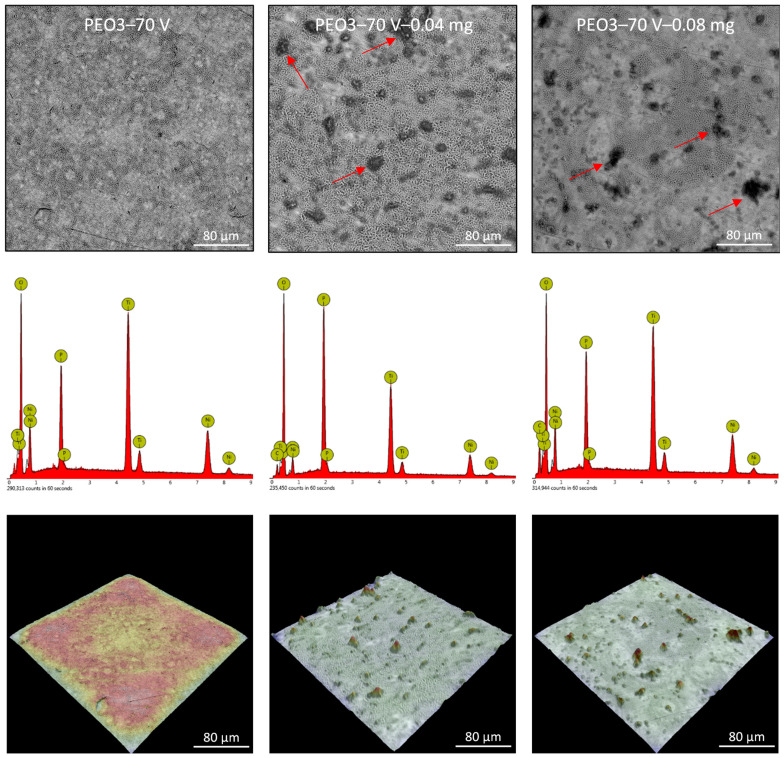
Surface morphology and chemical composition of the PEO3 sample, and PEO3 samples with nanodiamonds (0.04 or 0.08 mg); SEM—upper row; EDX—middle row; 3D reconstruction—lower row; red arrows—particles agglomerates.

**Figure 7:**
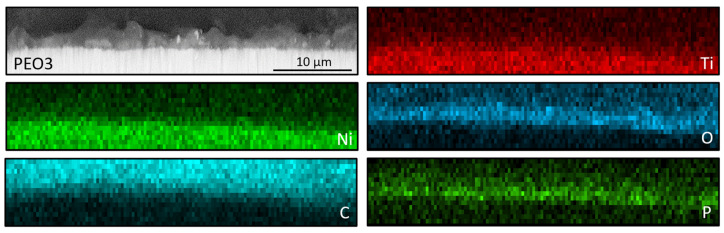
The exemplary cross section analysis and EDX mapping; PEO3 sample (70 V).

Plasma electrochemical oxidation is very often used to functionalize the surface of titanium and its alloys, in particular for biomedical applications. During this process, as a result of electrochemical processes, generated plasma, and high-temperature processes, a characteristic, porous oxide layer is formed. This layer is made up of substrate metal oxides and built-in electrolyte components. In the case of titanium and its alloys, diluted water electrolytes are the most often used, the use of which is practically impossible to modify the surface of the NiTi alloy. The PEO process of the NiTi alloy was carried out in concentrated phosphoric acid, which led to the formation of an oxide layer (Figure 4, Figure 5 and Figure 6: PEO1–PEO3). It is worth noting that in the case of this alloy, the PEO started at a voltage of 50 V, while for titanium it is about 90–100 V, depending on the process parameters. The 50 V caused a thin oxide layer with single pores to form on the NiTi surface (Figure 4), with visible scratches after the grinding process. Increasing the voltage to 60 V resulted in the appearance of characteristic pores for the PEO process (Figure 5). Interestingly, these pores were arranged in clear circles, which can be related to the formation of gas bubbles on the anode and the more intensive process at the boundary of the bubble–electrolyte–anode surface. The gas evolution is normal in the PEO process, but in aqueous solutions, the bubbles leave the reaction medium almost immediately. In the case of concentrated phosphoric acid, separation of the gas bubble is difficult because of the viscosity of the solution. Only the application of a higher voltage caused the entire surface of the alloy to be covered with a porous oxide layer (Figure 6). Regardless of the applied voltage, the oxide layer was composed of Ti, Ni, O, and P, which indicates that it could be a mixture of titanium and nickel oxides, as well as phosphorus incorporated from the electrolyte. The resulting layers had a thickness of less than 10 µm, and phosphorus was incorporated into the entire volume of the oxide coating (Figure 7).

The NiTi alloy, after the PEO process, was characterized by hydrophilic properties (Table 1). An increased value of the contact angle was characteristic of the sample oxidized at 70 V, which could be related to the gas trapped in the pores and the difficulty of their penetration by water. Interestingly, lower roughness was recorded for this sample, which may indicate homogenization of the surface.

The application of the ND-Aryl suspension to the NiTi alloy samples mostly resulted in partial covering of the characteristic oxide coating. Agglomerates of nanoparticles are visible on the surface, while a characteristic carbon peak appears in the EDX spectra (Figure 4, Figure 5 and Figure 6).

### 3.3. Biological Assessment

**Figure 8:**
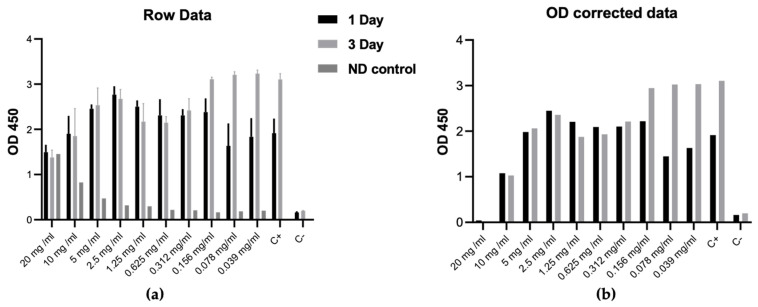
Cytotoxicity of ND examined by CCK-8 assay with human dermal fibroblasts during 3-days of co-cultivation. (**a**)—row data with ND cell-free control and (**b**)—data after the ND optical density correction. “OD 450”—optical density measured with 450 nm; “C+”—positive control (without ND), “C−”—cell-free control with cell media only.

To ensure the application of non-cytotoxic concentrations of ND for decoration of nitinol PEO coating, we evaluated the cytotoxicity profile starting from 20 mg/mL to 0.039 mg/mL. Our previous data demonstrated that some nanoparticles can influence metabolic assays [41] that lead to false positive results. The high concentration of nanoparticles in the cell culture medium can also increase the optical density (OD) during CCK-8 measurements and affect the final results. Figure 8a shows that the OD of the cell-free medium with ND at concentrations of 5–20 mg/mL has the same OD results as in the fibroblast group with ND. After correction for OD (Figure 8b), we clearly demonstrated that arylated ND exhibits cytotoxic effects at concentrations above 0.156 mg/mL. It should be noted that concentrations from 0.312 mg/mL to 2.5 mg/mL demonstrated inhibition of cell growth, while higher concentrations provide direct cell death. Taking into account these results, we chose the concentrations of 0.08 and 0.04 mg/mL as biocompatible for the decoration of nitinol PEO.

**Figure 9:**
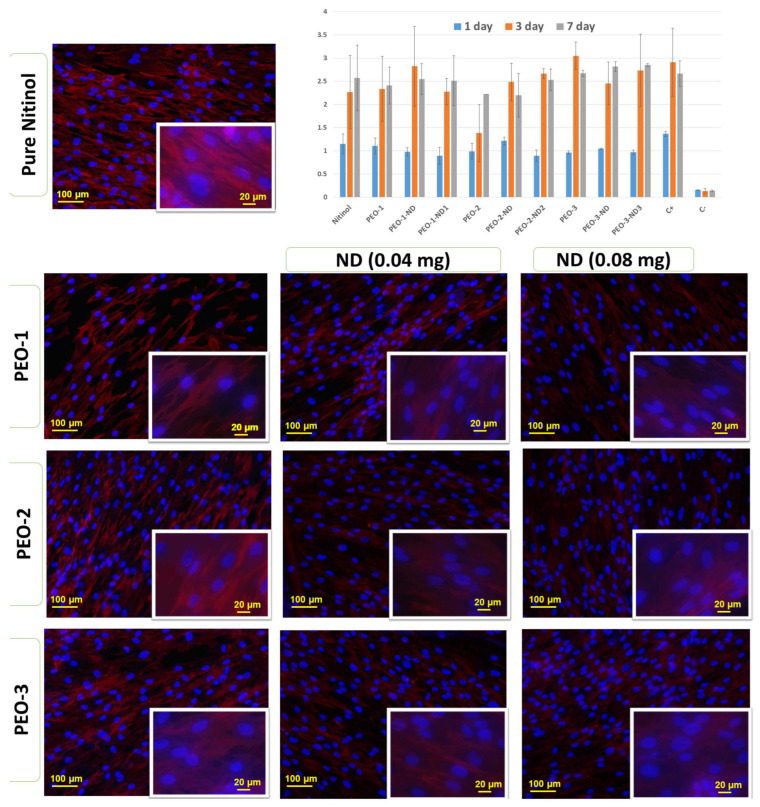
CCK-8 assay data on proliferation of human dermal fibroblast during the 7-day experiment (diagram) with fluorescent images of nuclei (blue) and cytoskeleton staining (red) on day 7 of cultures on metal NiTi samples. Where: PEO-1—50 V, PEO-2—60 V, PEO-3—70 V.

The uncoated nitinol surface demonstrated high biocompatibility and adequate adhesion of the fibroblast on day 1 after seeding with sufficient proliferation during the seven days of the experiment (Figure 9—CCK-8 assay diagram). We noticed a minor difference between the nitinol and positive control groups that could reflect the hydrophobic nature of the metal surface [42]. The PEO-1 and PEO-2 treatment regimens demonstrated better cell adhesion and increased fibroblast proliferation. As we demonstrated in our previous research, the large porous area of the modified surface and the decrease in the contact angle provided an appropriate environment for cell–surface interaction [43]. At the same time, the PEO-2 surface demonstrated significantly less cell adhesion on day 1 and insufficient proliferation dynamic up to day 7 of the experiment. The addition of ND did not affect cell adhesion and proliferation in the entire experimental group, but improved fibroblast proliferation in the PEO-2 group. These results demonstrated the safety of ND decoration and the positive influence on cell proliferation. ND functional groups can facilitate cell proliferation, as previously demonstrated for osteoblasts and vascular endothelial cells [24]. Fluorescent microscopy investigation (Figure 9) demonstrated that the PEO coating with and without ND decoration did not affect the fibroblast morphology as follows: on day 7 they have an elongated shape with oval nuclei and a well-developed cytoskeleton. Cells organized in a ‘cobblestone-like’ structure with aligned orientation. ND in both 0.04 mg/mL and 0.08 mg/mL did not affect cell morphology and orientation. 

## 4. Conclusions

Cardiovascular disease (CVD) remains a significant cause of mortality in Europe, with atherosclerosis being the primary underlying disease. Current treatment methods, such as statin drugs and BMS, have limitations in reducing plaque size and preventing restenosis. To address these challenges, this study introduces a novel approach to improve the performance of nitinol stents in the treatment of CVD.

Our research focuses on incorporating detonation nanodiamonds (DNDs) into a plasma electrolytic oxidation (PEO) coating on nitinol stents. DNDs, with their high surface-to-volume ratio, are functionalized and embedded within the oxide layer of the stents to simulate the function of high-density lipoproteins (HDLs) in facilitating reverse cholesterol transport (RCT). The characterization of the modified DND and PEO-coated nitinol surface confirms successful functionalization and stable suspension. Using the concentrated phosphoric acid as an electrolyte, we achieved the formation of an oxide layer with incorporated DND. The application of a higher voltage provides the formation of a porous layer that is composed of Ti, Ni, O, and P. The application of DND in the PEO solution leads to an increase in the hydrophilic properties that are favorable for biomedical applications. Biological assessments demonstrate the biocompatibility of the modified nitinol surface, revealing enhanced cell adhesion and proliferation. These findings offer a novel and promising approach to significantly improving the performance and long-term outcomes of nitinol stents in the treatment of CVD.

Using the unique properties of the DND and PEO coatings, this study opens new avenues for the development of highly effective and biocompatible nitinol stents that can address the limitations of existing treatments. These advances have the potential to greatly impact cardiovascular care and contribute to improved patient outcomes in the future.

## Figures and Tables

**Table 1:** Wettability and roughness of the NiTi samples.

Sample Label	Contact Angle, °	Roughness, µm
PEO1—50 V	28.01 ± 2.28	2.80
PEO2—60 V	27.95 ± 4.89	2.00
PEO3—70 V	80.85 ± 5.00	1.42
PEO1—50 V − 0.04 mg	26.00 ± 2.71	1.66
PEO2—60 V − 0.04 mg	31.91 ± 2.96	1.36
PEO3—70 V − 0.04 mg	28.68 ± 3.52	1.75
PEO1—50 V − 0.08 mg	31.66 ± 3.80	1.23
PEO2—60 V − 0.08 mg	24.74 ± 2.31	1.27
PEO3—70 V − 0.08 mg	37.37 ± 1.62	1.34

## Data Availability

The data are available by request.
